# Characteristics and Research Progress of Legume Nodule Senescence

**DOI:** 10.3390/plants10061103

**Published:** 2021-05-30

**Authors:** Shunxin Zhou, Chanjuan Zhang, Yi Huang, Haifeng Chen, Songli Yuan, Xinan Zhou

**Affiliations:** Key Laboratory of Biology and Genetic Improvement of Oil Crops, Oil Crops Research Institute, Chinese Academy of Agricultural Science, Ministry of Agriculture and Rural Affairs, Wuhan 430062, China; zhoushunrong@caas.cn (S.Z.); zhangchanjuan@caas.cn (C.Z.); huangyi@oilcrops.cn (Y.H.); zhouxinan@caas.cn (X.Z.)

**Keywords:** legume-rhizobium symbiosis, nodule senescence, N-fixation activity, cysteine proteases, abiotic factors

## Abstract

Delaying the nodule senescence of legume crops can prolong the time of nitrogen fixation and attenuate the lack of fertilizer in the later stage of legume crop cultivation, resulting in improved crop yield and reduced usage of nitrogen fertilizer. However, effective measures to delay the nodule senescence of legume crops in agriculture are relatively lacking. In the present review, we summarized the structural and physiological characteristics of nodule senescence, as well as the corresponding detection methods, providing technical support for the identification of nodule senescence phenotype. We then outlined the key genes currently known to be involved in the regulation of nodule senescence, offering the molecular genetic information for breeding varieties with delayed nodule senescence. In addition, we reviewed various abiotic factors affecting nodule senescence, providing a theoretical basis for the interaction between molecular genetics and abiotic factors in the regulation of nodule senescence. Finally, we briefly prospected research foci of nodule senescence in the future.

## 1. Introduction

Nitrogen is one of the most important limiting factors for the growth and development of plants in nature. At present, the high yield of crops largely depends on the use of nitrogen fertilizers. However, large-scale application of nitrogen fertilizers not only increases production costs but also causes environmental pollution. As a biological process between nitrogen-fixing microorganisms and legumes, symbiotic nitrogen fixation (SNF) directly converts atmospheric nitrogen into ammonia through nitrogenase [1]. Therefore, it is the most economical and environmentally-friendly way of achieving nitrogen fixation [2]. Moreover, SNF plays a significant role in enhancing the recovery of grassland ecosystems, promoting the growth of windbreak and sand fixation tree species, accelerating the remediation of heavy metal-contaminated land, improving the composition of soil nutrients, and ameliorating the agricultural weight loss and efficiency [3,4].

The formation of legume-rhizobium symbiosis is the result of mutual recognition between microorganisms and plants, as well as the interaction between signaling molecules [5]. First, flavonoid compounds released by the legume roots can induce rhizobia to produce Nod factors, which have the function as signal molecules and can be recognized by Nod factor receptors of legume plants. Then, the root hairs of legumes curl, deform and form infection threads [6] through which rhizobia penetrate the root tissue [7]. Simultaneously, some cortical cells of the root are stimulated to start dividing to form a primordial nodule [8]. The cells infected by the bacteria derive from root inner cortex cells, which differentiate and then proliferate to form the nodule meristem. The timing of initiation, development, and maturing of nodule organogenesis inside root cells has been well documented [9,10].

Nodules in legumes can be divided into two types depending on the persistence of the nodule meristem as follows: determinate nodules and indeterminate nodules, which have different characteristics in morphology [11]. Indeterminate nodules, such as those in *Medicago truncatula*, *Pisum sativum*, *Vicia faba*, and *Trifolium repens*, contain five different zones: the active meristem that allows the growth of the nodule (Zone I), the infection zone, where the cells differentiate and are infected by the bacteria (Zone II), the nitrogen fixation zone (Zone III), in which bacteroids fix atmospheric nitrogen, and the senescent zone (Zone IV), where bacteria and plant cells are deconstructed, accompanied by the destruction of symbiosis [12]. Determinate nodules, such as *Glycine max* and *Lotus japonicus,* have non-persistent meristems, and all the infected cells are always at the similar stage of development. In both determinate and indeterminate nodules, rhizobia supply nitrogen sources for the growth and development of legume plants by SNF, and in return, legumes provide carbon sources to rhizobia to sustain their growth and reproduction within the nodules [13].

There are three major approaches to increase SNF efficiency: increasing the number of nodules to improve nitrogen fixation efficiency; improving nitrogen-fixing enzyme activity, and delaying nodule senescence to extend the time of nitrogen fixation and increase nitrogen fixation amount. Among them, delaying nodule senescence and prolonging the time of nitrogen fixation are the key measures to ensure nitrogen fixation and adequate supply of nitrogen to legumes in the reproductive period and to solve the problem of late defertilization in legumes, which often occurs in pod stage when the symbiotic nitrogen fixation ability of legume crops is gradually weakened, and meanwhile it is hard to artificially supply extra nitrogen fertilizer to meet the demand at this stage. In the present review, we summarized the characteristics and research methods of legume nodule senescence, and the research progress related to molecular and abiotic factors in the regulation of nodule senescence. In our current work, we aimed to compile information on legume nodule development and senescence and to provide ideas to further delay nodule senescence.

## 2. Characteristics and Research Methods of Legume Nodule Senescence

Generally, the efficiency of nitrogen fixation in legume plants reaches a peak at 4–6 weeks after inoculation with rhizobia, after which the bacteroids differentiate and the nitrogen fixation ability is gradually weakened [14]. Nodule senescence is a complex and programmed process, including the reduction of Leghemoglobin content, structural changes of nodule cells, the decrease of nitrogenase activity, changes in the lifestyle and growth capacity of bacteria, and so on. The morphology, physiological and biochemical changes and the corresponding methods are summarized in Table 1.

During the nodule senescence, in particular, the color of the nitrogen fixation zone is converted from pink to green due to the nitration of the heme group of functional leghemoglobin (LHb) [15]. This nodule color change is caused by nitrogen biomobilization and a rapid decrease in LHb content [16]. Therefore, we can assess the degree of nodule senescence by calculating the ratio of senescent nodules to the total nodules by observing the changes in nodule color, and detect the content of LHb by using the cyanmethemoglobin method, which is a rapid, quantitative method for the determination of LHb in legume nodules [17,18]. Besides, we can also examine the content of LHb at the transcriptional and protein levels by using qPCR and Western blotting analysis.

The most representative characteristic of nodule senescence is the structural changes of the nodule cells, including progressively reduced electron density, the appearance of numerous vesicles in the cytoplasm, increased number of peroxisomes, the formation of complex elongated structures by mitochondria, symbiotic membrane disintegration, damaged cell wall, and lysis of bacteroids [14,19,20]. Generally, paraffin sections of nodules are made to observe the dynamic changes of senescence zone [21], the TdT-mediated dUTP-biotin nick end-labeling (TUNEL) staining assay is used to detect the programmed cell death or apoptotic-like cell death in nodules [22,23], transmission electron microscopy is utilized to observe the morphology of the inner cells, the electron density of the symbiosis, and the integrity of the symbiotic membrane [23], and in situ live/dead staining assay has been found to detect the rhizobia with intact cell membranes or damaged membranes [24,25].

**Table 1:** The morphology, physiological and biochemical changes of legume nodule senescence and the corresponding detection methods.

Characteristics of Nodule Senescence	Corresponding Detection Methods	Reference
**Morphology changes**		
The color of nodules is converted to green; loss of turgidity in nodules	Calculating the ratio of senescent nodules to the total nodules	[15]
Less electron dense; appearance of numerous vesicles and peroxisomes;	Nodule paraffin sections micromorphology analysis; electron microscope scanning	[14,19,20]
The formation of complex elongated structures by mitochondria		
Symbiotic membrane disintegration; damaged cell wall and lysis of bacteroids	Nodule paraffin sections micromorphology analysis; electron microscope scanning;TUNEL staining assay; in situ live/dead staining assay	[14,19,20,24,25]
**Physiological and Biochemical changes**		
Decreased nitrogenase activity	Acetylene reduction assay (ARA)	[26,27]
Decreased leghemoglobin content	The cyanmethemoglobin method, qPCR and Western blotting analysis	[17,18]
Decreased lifestyle and growth capacity of Rhizobium	Testing the lifestyle and growth capacity of Rhizobium	[28,29]
Increased the concentration of nitric oxide (NO), ethylene, ABA and so on	Measuring the nodule senescence-related metabolites and hormone signals	[5,30]
Increased in the expression of nodule senescence-related marker genes	Laser microdissection; ACC immunolocalization; qPCR	[31]

Decreased nitrogenase activity, lifestyle and growth capacity of bacteria are also the major features of nodule senescence [28,29]. The decline in nitrogen-fixing enzyme activity is measured using the acetylene reduction assay (ARA), which is a high sensitivity, rapid, low cost, and nondestructive method [26,27]. Rhizobium is an important player in nodule senescence, and the morphological and physiological characteristics of senescent nodules have revealed that half of the rhizobia surviving in a hemibiotrophic-like lifestyle cannot grow [28,29]. Therefore, testing the lifestyle and growth capacity of bacteria is also an efficient method to evaluate nodule senescence.

Legumes can control the formation of symbiosis and the lifespan of nodules through the molecular dialogue between metabolites and hormones [5,30]. Therefore, nodule senescence can be detected by measuring the metabolites and hormone signals that determine the onset of nodule senescence in plants [32]. For example, endogenous ethylene plays a role in the formation and position of nodule primordia [33,34,35].Two ethylene inhibitors silver ions (Ag+) and aminoethoxy-vinylglycine (AVG) can counteract the interfering effect of ethylene, we therefore measured the position of nodule formation in the presence of AVG or Ag + to test whether the endogenous ethylene provided the position information to control the formation of nodule primordia [35].Furthermore, according to known molecular markers associated with nodule senescence, several methods such as laser microdissection, qPCR, and ACC immunolocalization are employed for nodule senescence detection [31].

In summary, the morphology, physiological and biochemical characteristics of legume nodule senescence and the corresponding detection methods have been described in detail. According to these findings, we can easily identify the nodule senescence phenotype of legumes and provide technical support for functional studies of genes in nodule senescence.

## 3. Gene Regulation Related to Legume Nodule Senescence

**Figure 1:**
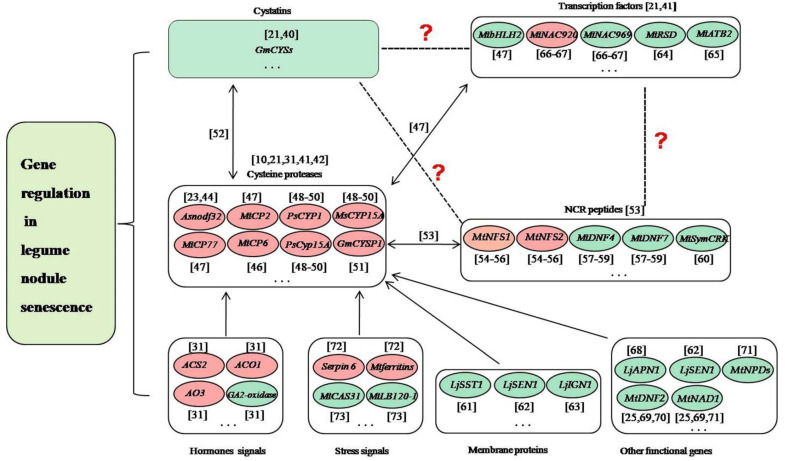
Prediction of the relationships among cysteine proteases, cystatins, NCR proteins, transcription factors and other genes in the regulation of nodule senescence. The dotted lines and/or question marks indicate that the relationship between the two proteins is uncertain. The double arrows indicate that the direct regulatory relationship between the two proteins is proved. The single arrows indicate that the relationship between the two proteins exists. The red ellipses indicate that the genes positively regulate nodule senescence. The green ellipses indicate that the genes negatively regulate nodule senescence. The ellipsis indicates that many genes have not yet been identified [10,21,23,25,31,40,41,42,44,46,47,48,49,50,51,54,55,56,57,58,59,60,61,62,63,64,65,66,67,68,69,70,71,72,73].

Delaying nodule senescence and/or prolonging nitrogen fixation time are the key measures to solve the problem of late defertilization of legumes, which is critical for the planting of legumes and agriculture. Nodule senescence is a genetically controlled process, and transcriptomic studies of the model legumes *L. japonicus* and *M. truncatula* have identified lots of up- or down-regulated genes in senescent nodules [19,36,37,38]. In soybean, we analyzed the nodule development at five different developmental stages (branching stage, flowering stage, fruiting stage, pod stage and harvest stage), and found many nodule senescence-related genes, including those encoding soybean cysteine proteases, cystatins, cysteine-rich proteins and transcription factors, as well as proteins involved in plant-pathogen interactions [21]. We performed whole-genome surveys of soybean papain-like cysteine protease (PLCP) family genes [39], soybean cystatin family genes [40] and soybean C2H2 zinc finger family genes [41], analyzed their expression profiles during nodule development and senescence, and identified dozens of candidate genes related to nodule senescence. Besides, many genes have been studied to play roles in regulating the nodule senescence of legume plants in recent years. These genes mainly include cysteine proteases, transcription factors, peptides, enzymes, and other functional genes (Figure 1).

During the progress of nodule senescence, the expressions of various hydrolases are up-regulated to trigger large-scale protein degradation, which is a hallmark of nodule senescence, especially for cysteine proteases [42]. Cysteine proteases are a class of proteins widely involved in the senescence and programmed cell death of various tissues and organs of plants [43], and they have been shown to play key roles in legume nodule senescence [10,31]. In *Astragalus sinicus*, *Asnodf32* is a nodule-specific cysteine protease and RNAi interference with the expression of *Asnodf32* delays nodule senescence and prolongs nodule lifespan [23,44]. In *M. truncatula*, Mtcp1-Mtcp6, a conserved cysteine protease subfamily, may be involved in the degradation of symbiont structure [45], and *MtCP6* promotes nodule senescence [46]. *MtCP77* positively regulates nodule senescence by accelerating plant PCD and reactive oxygen species (ROS) accumulation [47]. In pea, *PsCYP1* is expressed at the onset of senescence in the indeterminate nodules, and inhibition of *PsCyp15A* or *MsCYP15A* delays the senescence of nodules [48,49,50]. In soybean, *GmCYSP1* may participate in nodule development and senescence [51]. Transcriptomic studies have shown that dozens of papain-like cysteine proteases (*PLCPs*) are associated with nodule senescence [21,52]. We performed the genome-wide survey of *PLCPs* to detect their expression in root nodule symbiosis, and identified many nodule senescence-related *PLCPs* [39]. Moreover, cysteine protease can specifically inhibit nodule cysteine-rich (NCR) peptides, which mainly exist in legumes and are closely related to the terminal differentiation of rhizobia [53]. For example, *MtNFS1* and *MtNFS2* can induce bacterial death and early nodule senescence in rhizobial strain-dependent manner [54,55,56]. *MtDNF4* and *MtDNF7* are essential for bacterial survival, bacterial differentiation and nitrogen fixation [57,58,59]. *MtSymCRK* can prevent nodule senescence and avoid defense-like reactions [60]. Cysteine protease can be inhibited by its natural inhibitor cystatin, several of which are differently expressed in symbiosis and play roles in nodule senescence [21,40,52]. Cysteine protease can also be regulated by transcription factors during nodule senescence. For example, *MtbHLH2* can repress *MtCP77* to negatively regulate nodule senescence [47]. *MtNAC920* can up-regulate *MtCP2* to play positive roles in nodule senescence [47]. These findings suggested complex relationships may exist among cysteine proteases, cystatins, NCR proteins and transcription factors. In the regulation of the root nodule senescence of legumes, previous studies have proved that cystatins and transcription factors can regulate cysteine proteases, cysteine proteases can inhibit NCR proteins, while whether transcription factors can regulate cystatins and NCR proteins, and whether cystatins can regulate NCR proteins remain unclear (Figure 1).

Nodule senescence is coregulated by legumes and rhizobia, and a series of cellular metabolic processes and the transport of nutrients are involved in this process. In legume plants, except for cysteine proteases, the genes associated with these biological processes mainly are membrane proteins, transcription factors and other functional genes. *LjSST1*, which is a nodule-specific sulfate transporter that is located on the symbiosome membrane in Lotus nodules, is critical for symbiotic nitrogen fixation [61]. *LjSEN1*, encoding an integral membrane protein, plays important roles in regulating the nitrogen fixation activity and the differentiation of bacteroids or symbiosomes [62]. *LjIGN1* is an ankyrin-repeat membrane protein and is essential for bacteroid differentiation and functioning [63]. Many soybean C2H2 transcription factors may play key roles in nodule senescence [41]. *MtRSD*, which encodes a nodule-specific C2H2 transcription factor, can repress the transcription of *VAMP721a* and promote symbiosome development in *M.truncatula* [64]. A bZIP transcription factor *MtATB2*, which can regulate carbon metabolism and metabolite partitioning, is regulated by sucrose and enhanced during nodule senescence [65]. Two NAC transcription factors *MtNAC969* and *MtNAC920* can be induced by root abiotic stress and have been shown to play roles in nodule senescence [66,67]. bHLH2 transcription factor *MtbHLH2* plays significant roles in plant PCD, ROS accumulation, and nodule senescence [47]. *LjAPN1* is a nepenthesin-type aspartic peptidase, and plays critical roles in the persistence of nitrogen-fixing symbiosis functioning in a rhizobial strain-dependent manner [68]. *MtNAD1* and *MtDNF2* are essential for the maintenance of rhizobial endosymbiosis and the control of plant defense during the symbiotic colonization [25,69,70]. Lines with different *MtNPDs* mutation combinations presented smaller nodules, earlier nodule senescence, or ineffective nodules [71].

Nodule senescence can also be regulated by the genes associated with phytohormones and stress. Two genes *ACS2* and *ACO1*, which encode two enzymes that are involved in the biosynthesis of ethylene, can activate nodule senescence [31]. Similarly, aldehyde oxidase AO3, which is a key enzyme for the biosynthesis of ABA, can also positively regulate nodule senescence [31]. Opposite to the above-mentioned three genes, GA2-oxidase, which is involved in the catabolism of gibberellins, has been shown to suppress nodule senescence [31]. Mt serpin6 and Mtferritins can mediate ordered drought-induced senescence and reduce plant growth [72]. MtCAS31 protects MtLb120-1 from the damage of drought stress to delay nodule senescence [73].

In this part, we outline the key genes currently known to be involved in the regulation of nodule senescence, mainly including cysteine proteases, transcription factors, cystatins, NCR peptides, hormones signals-related genes, stress signals-related genes, membrane proteins and other functional proteins (Figure 1). These findings offer molecular genetic information for breeding varieties with delayed nodule senescence. Cysteine proteases usually are considered as adequate molecular markers of nodule senescence [31], and the relationships between the cysteine proteases and other key genes are universal (Figure 1), while the regulatory mechanism of cysteine proteases and the coordinated regulation of cysteine proteases and other key genes during nodule senescence should be the focus in the future works.

## 4. Abiotic Factors in the Regulation of Nodule Senescence

Nodule senescence is mainly triggered by normal development, while environmental stress can also affect and regulate its progress [45,74,75], such as drought [72,76], salt [66,77], darkness [45,78], nitrate [37,79,80,81], and so on. Under drought stress, more deleterious reactive oxygen species (ROS) are produced, and the synthesis of ferritin is inhibited, thus accelerating the senescence of root nodules [72], while leaves of low photosynthetic capacity are sacrificed in favor of nodule nitrogen metabolism to delay nodule senescence [76]. Salt stress can inhibit the N2-fixation activity and nodule respiration, and induce premature senescence of existing nodules [77]. Nitrogenase activity and the content of leghemoglobin are decreased under prolonged dark treatment, which can rapidly trigger a wide range of nodule senescence [45]. In nitrate-induced nodule senescence, the antioxidant content and nitrogenase activity are reduced, leading to serious impairment of nodule metabolism related to nodule senescence [79]. Besides, it has also been shown that the expression of *acds* is increased in nodules under hypoxic conditions, resulting in postponed degradation of enzymes associated with nitrogen fixation and delayed senescence of nodules [82].

Accumulating evidence suggests that oxidative damage exists in legume nodule senescence [14,78,83,84], and in these oxidative stress-induced aging nodules, ROS (mainly organic peroxides), oxidized glutathione and homoglutathione, catalytic Fe, lipid peroxidation, and oxidatively modified proteins and DNA bases are increased [84,85,86]. Oxidative stress is a result of over production of free radicals and is associated ROS [87]. ROS may induce irreversible posttranslational modification (carbonylation, advanced glycation) of crucial proteins in nodules, which play key roles in cell metabolism and nodule senescence [88]. Mitochondria are an important source of regulatory redox signals and an early target of oxidative modifications in senescing legume nodules [14,89]. ROS (such as H2O2) was shown to act as signaling molecule that regulate nodule senescence [14,32], and the concentrations of ROS will be tightly controlled by antioxidant enzymes and metabolites [90]. The antioxidant systems in symbiotic nodules can protect cells from oxidative damage [14,91,92], and the roles of ROS and antioxidant systems in nodule senescence have been reviewed comprehensively elsewhere [14,90].

As a signaling and defense molecule involved in diverse plant developmental processes and the plant response to pathogens, nitric oxide (NO) has been detected as a signal in nodule senescence [93]. Reducing the NO concentration can delay the senescence of nodules in dark conditions, while increasing the endogenous NO leads to cytological modifications of the nodule structure and the early expression of a specific senescence marker [93]. Meanwhile, the rhizobia can control NO-mediated post-translational modifications of legume proteins to regulate nodule senescence [93,94,95]. *Hmp*-a, encoding a NO detoxification enzyme, can inhibit the level of NO and delay nodule senescence [93,96]. Similarly, *LjGlb1-1*, a non-symbiotic class 1 Hb, can negatively regulate the level of NO, enhance the nitrogenase activity of mature and senescent nodules, and delay nodule senescence [97,98]. Meanwhile, iron-induced NO leads to an increased expression of ferritin during the senescence of *L. japonicus* nodules, supporting the above-mentioned point [99].

Nodule senescence is also tightly regulated by plant hormones, which are small molecules playing versatile roles in regulating plant growth, development, and responses to the environment [100]. These nodule senescence-related hormones mainly include ABA, ethylene, gibberellins, and jasmonate (JA). ABA plays roles in plants generally by decreasing growth rate and enhancing cell sustainability, while it also can inhibit lateral root development [101]. ABA is involved in the response of roots to high nitrate levels in the soil [102], and regulates the root nodule senescence [103]. When legumes are treated with ABA, the contents of leghemoglobin and N-fixation are declined, leading to the premature senescence of root nodules [103]. During nodule senescence, ethylene response factors (ERFs), SAM synthetase, ACC synthase, and ACC oxidase are up-regulated, which can increase ethylene production and activate nodule senescence [104,105]. Besides, when ethylene biosynthesis and signaling are altered, the bacteroid number per symbiosome [106], bacterial elongation [107] and bacteroid senescence will change [108]. Gibberellins are a large group of diterpenoid carboxylic acids in higher plants and have been well studied in nodulation, nodule development, and senescence [109,110,111]. The size of the nodule and the zone of nitrogen fixation are increased, and the number of nodules and senescence zone is decreased in the GA3-treated legumes [111], suggesting a negative effect of GAs on the nodule senescence. Furthermore, GA3 treatment can stimulate nodule meristem bifurcation [111], indicating a possible role of GAs in the redifferentiation of nodule meristem. JAs are important molecules in regulating physiological processes in plant growth and development, which can mediate the plant responses to biotic and abiotic stresses [112]. JAs are highly expressed in root nodules, and previous studies have shown that JAs can inhibit the expressions of early nodulation genes to negatively regulate nodulation [113]. Besides, they can also affect nodule cell growth and senescence process by affecting the antioxidant metabolism [114].

**Figure 2:**
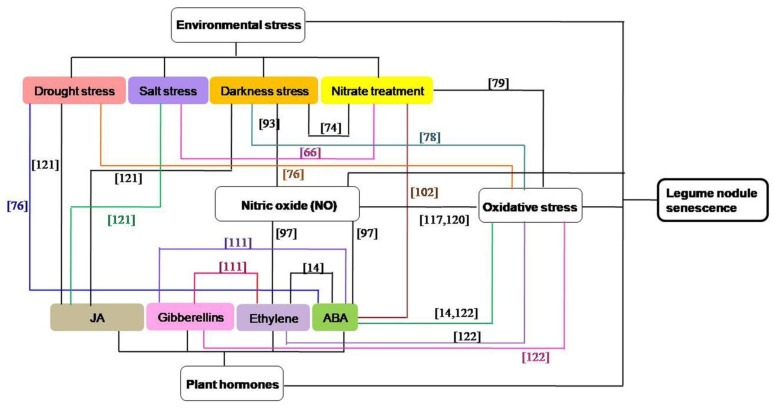
The interaction network of different abiotic factors in the regulation of the senescence of nodules [14,66,76,78,79,93,97,102,111,117,120,121,122].

Among the above-mentioned factors, the concentration of NO in legumes will increase when the plants are exposed to environmental stresses [115,116,117]. NO may be produced downstream in the phytohormone signaling pathway in nodules [97], and plant hormones play critical roles in plant responses to adverse environmental conditions [100,118]. ROS and antioxidants can interact with hormones in the orchestration of nodule senescence [14], ROS and antioxidant systems are also participate in environmental stress-induced nodule senescence [76,78,79,119], and crosstalk between ROS and NO appears to be a key point to understand the redox regulation of symbiosis [120]. Therefore, these four signaling pathways can interact with each other in the regulating the senescence of nodules, and the interactions between different hormones or different environmental stresses are also existed during nodule senescence (Figure 2). In darkness-induced nodule senescence, the degree of senescence is reduced by removing NO from the nodules, suggesting that NO could be an intermediate in this process [93]. ABA and ethylene can induce NO accumulation during nodule senescence and the senescence-associated changes are suppressed by the NO scavenger cPTIO, indicating that ABA and ethylene trigger nodule senescence maybe by regulating the NO levels of nodules [97]. JAs, which have a strong senescence-promoting effect, were recognized as being signals in plant responses to lots of biotic and abiotic factors, including salt stress, drought stress and darkness stress [121]. ABA can interact with drought stress [76] or nitrate treatment [102] in the regulating the nodule senescence. The interactions between different hormones [14,111] or different environmental stress factors [66,74] are also found during nodule senescence. In drought stress [76], darkness stress [78] and nitrate treatment- included [79] nodule senescence, ROS and oxidatively modified proteins are increased, and these oxidative damage may have originated from the decrease in antioxidant defenses. Ascorbate, an important nodule antioxidant, is involved in dioxygenase reactions, such as those required for the synthesis of ABA, gibberellic acid and ethylene [14,122]. Both ROS and NO can mediate post-translational modification of proteins during nodule senescence, and legume plants possess different hemoglobins which play a significant role in ROS and RNS metabolism by contributing to ROS production and NO scavenging [117,120].

Here, we analyzed the effects of common environmental stresses, oxidative stress, NO, and plant hormones on the senescence of nodules, and reviewed the interactions between these four signaling pathways in regulating nodule senescence. Future research on screening or cultivating legumes and/or rhizobia those are resistant to these abiotic factors, and discovering additional crosstalk mechanisms among these signaling pathways will be important themes in the field of nodule senescence research.

## 5. Summary and Prospect

Delaying the senescence of root nodules and prolonging the time of nitrogen fixation are key measures to solve the lack of fertilizer in the later stage of legume crop cultivation. These approaches are effective ways to fully utilize the role of nitrogen fixation and promote the sustainable development of green agriculture. However, the means by which to delay nodule senescence and prolong the time of nitrogen fixation remain to be further studied. 

In the present review, the structural and physiological characteristics of legume nodule senescence were described in detail, and several effective research methods detecting the senescence of nodules were summarized. However, the molecular mechanism of nodule senescence is still unclear. Although a large number of mutations of legume plants have phenotypes associated with nodule senescence, methods regarding the identification of genes that precisely control nodule senescence have not yet been well established. Additionally, many cysteine proteases, transcription factors, peptides, enzymes involved in the biosynthesis of hormones, and other functional genes for nodule senescence have been identified, although the regulatory mechanism of these key genes remains unclear. Therefore, the coordinated regulation of various key genes during nodule senescence should be the focus of future works.

Abiotic stresses are a major agricultural restriction factor affecting crop yield. Meanwhile, they also play key roles in regulating the root nodule senescence. Here, we reviewed the effects of common environmental stresses, oxidative stress, NO, and plant hormones on the senescence of nodules. Environmental stress factors such as drought, salt, darkness, and nitrate mainly reduce the activity of nitrogen fixation and promote the senescence of root nodules. Therefore, it is important to enhance the resistance of symbiosis to environmental stress to delay the nodule senescence. Moreover, screening or cultivating legumes and/or rhizobia that are resistant to these environmental stress factors is the subsequent focus. The redox regulation of nodule senescence mainly depends on ROS and antioxidant systems, and the roles of ROS and antioxidant systems in nodule senescence have been reviewed comprehensively, while the mechanism of the balance between ROS and antioxidant system is unclear. NO, as one of the RNS (reactive nitrogen species) involved in the interaction between legumes and rhizobia, is an important factor affecting nodule senescence, and it has been detected as a signal in nodule senescence. However, the mechanism underlying the NO-regulated nodule senescence is still unknown. Some plant hormones, including ABA, ethylene, gibberellins, and JA, also play important roles in the regulation of nodule senescence, while little is known about the regulatory mechanisms of these hormones and interactions between different hormones during nodule senescence. Additionally, ROS or NO can interact with hormones and environmental stress factors, and hormones can interact with environmental stress factors. Therefore, the coordinated regulation of various factors in nodule senescence will be very vital in future work.

In summary, we reviewed the structural and physiological characteristics and related research methods of legume nodule senescence, as well as important genes and abiotic factors related to legume nodule senescence. Our present review provided theoretical guidance for the cultivation of high nitrogen fixation legume and rhizobium varieties, and clues for establishing the technology of delaying nodule senescence, prolonging effective nitrogen fixation time, and enhancing SNF effect in legume-rhizobial symbiosis.
